# Factors associated with grief in informal carers of people living with Motor Neuron Disease: A mixed methods systematic review

**DOI:** 10.1080/07481187.2023.2191351

**Published:** 2023-03-30

**Authors:** Ana Paula Trucco, Tamara Backhouse, Eneida Mioshi, Naoko Kishita

**Affiliations:** School of Health Sciences, University of East Anglia, Norwich, UK

## Abstract

The purpose of this mixed methods systematic review was to identify factors associated with anticipatory grief, post-death grief, and prolonged grief in informal carers of people living with Motor Neuron Disease (MND) to inform future research and practice. Six electronic databases were searched and two quantitative and eight qualitative studies were identified. Five overarching themes were generated through thematic synthesis. The findings suggest that there are factors that may affect different grieving processes. It might be particularly important to target some factors prior and after the death of the person living with MND such as the knowledge about the progression of the disease, changes in relationships, anxiety and depressive symptoms of carers, and planning for death of the care recipient. Factors that may affect all three grieving processes were also identified such as negative experiences of caregiving, experiences of losses, end of life and psychological support, and emotional avoidance coping.

## Introduction

Informal carers (e.g., family members) of people living with Motor Neuron Disease (MND) are at a high risk of experiencing clinically significant levels of grief. MND, also known as amyotrophic lateral sclerosis (ALS) or Lou Gehrig’s disease, is a neurodegenerative, terminal disorder which leads to progressive muscle paralysis, the inability to swallow and speak, and respiratory failure (Kiernan et al., 2011). The average life expectancy of people living with MND after diagnosis is 2–3 years (Shaw et al., 2014). Given the rapid and progressive nature of MND, informal carers are often confronted with a series of loss experiences.

Grief has been defined as “the reaction to the perception of loss with symptoms including yearning, sadness, anger, guilt, regret, anxiety, loneliness, fatigue, shock, numbness, positive feelings, and a variety of physical symptoms unique to the individual” (Rando, 2000). Grieving processes for informal carers of people living with MND can start before the care recipient’s death (anticipatory grief), which are linked to the present changes and losses that carers experience during the trajectory of the disease. When distressing grieving experiences remain untreated following the death of the care recipient (post-death grief) they can lead to prolonged grief disorder (PGD), which involves severe, pervasive, and persistent grief reactions after the death of a person, causing distress and resulting in functional impairment in daily life (Prigerson et al., 2009). PGD can be diagnosed 6–12 months following the loss of a person according to the International Classification of Disease-11 (World Health Organization, 2019) and the Diagnostic and Statistical Manual of Mental Disorders-5 (American Psychiatric Association, 2022). The intensity of grief reactions after a loss will gradually decrease in most people and the bereaved person will be able to manage and integrate grief (Lundorff et al., 2017; Szuhany et al., 2021). Nevertheless, a significant minority of bereaved people will develop PGD with a pooled prevalence of 9.8% in the adult bereaved population (Lundorff et al., 2017). Informal carers of people living with MND are potentially at a greater risk of grieving more intensely beyond the period typically considered normal (O’Brien et al., 2016) with a prevalence of 49.6% and 8.7% of being at moderate and high risk of developing PGD respectively (Aoun et al., 2020). Moreover, carers of people living with MND are suspectible of poor mental and physical health (Aoun et al., 2013; Bergin & Mockford, 2016; Pagnini, 2013), which are considered to be linked to PGD (Lenger et al., 2020).

Currently, evidence on interventions that can effectively target grief processes among informal carers of people living with MND is scarce. Some systematic reviews on interventions for individuals who have experienced the death of a family member with other conditions reported that interventions targeting grief, such as psychotherapy and counseling, have a positive effect on alleviating grief symptoms (Currier et al., 2008; Johannsen et al., 2019; Wilson et al., 2017). Moreover, studies focusing on interventions targeting PGD, such as group psychotherapy (Rosner et al., 2011) and cognitive behavior therapy, have proved to be effective in the general bereaved population (Rosner et al., 2014, 2015), which may also work for informal carers of people living with MND. However, there are no studies reporting if these interventions improve bereavement outcomes in this population and no guidance for how grieving processes should be treated in informal carers of people living with MND. Understanding factors affecting different grieving processes, specifically in informal carers of people living with MND, is critical to inform future research and practice.

There are some existing systematic reviews focusing on factors affecting psychological outcomes in family carers of people living with MND such as burden, depression, anxiety, and quality of life (Aoun et al., 2013; Gluyas et al., 2017). Other systematic reviews are focused on factors contributing to positive and negative carers’ experiences of caring (Holkham & Soundy, 2018) and the need for palliative care (Flemming et al., 2020). However, there are no systematic reviews that have specifically looked at factors affecting grief in this population.

There are existing systematic reviews that focused on factors affecting grieving processes in informal carers of other populations (Chan et al., 2013; Crawley et al., 2022; Mason et al., 2020; Sanderson et al., 2022). These reviews were focused on family carers of people living with dementia (Chan et al., 2013; Crawley et al., 2022), patients in intensive care unit settings (Sanderson et al., 2022) and the bereaved general population (Mason et al., 2020). Findings from these studies reported that poorer carer psychological and physical health, cohabitation with the care recipient prior to institutionalization (Chan et al., 2013; Crawley et al., 2022), and dysfunctional copying styles (e.g., self-blaming) (Crawley et al., 2022) were associated with increased anticipatory grief symptoms. On the contrary, pre-morbid marriage satisfaction and the provision of more social support decreased anticipatory grief symptoms (Crawley et al., 2022). Greater post-death grief symptoms were related to being a spouse carer and carer pre-death depression (Chan et al., 2013). The strongest predictors of PGD in family carers of non-MND care recipients were found to be being a spouse carer (Chan et al., 2013; Sanderson et al., 2022), living alone following the death of the care recipient (Sanderson et al., 2022), high levels of carer pre-death depression (Chan et al., 2013), how the death occurred (Mason et al., 2020; Sanderson et al., 2022), poor psychological and physical health in carers, negative perceptions of social support, and facing multiple deaths (Mason et al., 2020).

Although some factors identified in previous reviews may be relevant to informal carers of people living with MND, MND carers’ experiences can be different from carers of people living with other conditions due to the relative rarity of the disease, the rapid and progressive escalation of symptoms, the increasing personal assistance and care required, and a rather short life expectancy. Furthermore, some of the risk factors identified in the previous studies in non-MND carer populations may not be relevant to carers of people living with MND such as multiple deaths. Therefore, it is important to bridge this gap in the current literature to inform future interventions aimed at grieving informal carers of people living with MND. The aim of this systematic review was to identify factors associated with anticipatory grief, post-death grief, and PGD specific to informal carers of people living with MND.

## Methods

This systematic review was designed and reported according to Preferred Reporting Items for Systematic Reviews and Meta-Analyses (PRISMA) guidelines (Page et al., 2021). The protocol was registered with the International Prospective Register of Systematic Reviews (PROSPERO registration number: CRD42021292798).

### Eligibility criteria

The Population, Intervention, Comparison, Outcomes and Study (PICOS) model was used as a framework to formulate the eligibility criteria. Due to the nature of our research question, the Intervention and Comparison categories were not applicable for this systematic review.

#### Study design

Articles published in peer-reviewed journals and in English, Spanish, or Portuguese were included. Cross-sectional, longitudinal, and qualitative studies were eligible. For mixed-methods studies, eligibility criteria were applied to the quantitative and qualitative components separately. We excluded dissertations, theses, book chapters, interventional studies, and systematic reviews studies.

#### Population

Studies were eligible if they recruited informal carers of people living with MND. Informal carers included both current carers (those currently providing care) and former (bereaved) carers. When a study included both informal and formal carers or included informal carers of people living with other conditions, the findings had to be reported separately for informal carers of people living with MND to be eligible.

#### Outcome

Any type of grief experiences occurring before and after the death of the person living with MND were eligible (anticipatory, post-death, and PGD). Quantitative studies were eligible if they used a standardized measure of grief and explored associations between grief and other variables. Qualitative studies were eligible if they aimed to explore grieving experiences of carers using interviews or an analysis of answers to free-text survey questions. Qualitative studies that aimed to explore MND caregiving experiences in general were also eligible if they included questions explicitly asking about grieving experiences.

### Information sources and search strategy

A systematic literature search of published studies was conducted using the electronic databases Scopus, MEDLINE, CINAHL and PsycINFO for English articles and SciELO and LILACS for Spanish and Portuguese studies. Sources were searched from database inception to 21st November 2021. An additional hand search of reference lists of included studies and relevant published systematic reviews on grief in informal carers was carried out. The search strategy included terms such as grief, informal carers and MND. The complete search strategy for each database is available in supplementary materials (see supplementary Table 1).

**Table 1.** Characteristics of included studies.

		Carer type	Place of recruitment	Carer gender	Mean age (*SD*)	Relationship	Type of grief	Validated measure of grief used
First author (year)	Country	Sample size		(Women %)	Age range	(Spouse %)		
Quantitative (cross-sectional) studies								
Aoun, Cafarella et al. (2021)	Australia	Former	MND Associations	73.8	63.1 (12.7)	72.2	Post-death	PG-13
		*n* = 393			22–91 years		Prolonged	
Aoun et al. (2020)	Australia	Former	MND Associations	73.0	63.5 (12.3)	73.3	Prolonged	PG-13
		*n* = 393			22–91 year			
Qualitative (individual interview) studies								
Aoun et al. (2012)	Australia	Former	MND Associations	81.3	65.3 (10.3)	100.0	Post-death	PG-13
		*n* = 16			NR		Prolonged	
Aoun, Noonan, et al. (2021)	Australia	Former	MND Associations	73.0	63.5 (12.3)	73.7	Post-death	PG-13
		*n* = 393			22–91 years		Prolonged	
Poppe et al. (2022)	Switzerland	Former	ALS centers	96.0^a^	NR	NR	Post-death	NA
		*n* = 14			28–74 years^a^			
Ray and Street (2007)	Australia	Current	MND Association	58.3	NR	70.8	Anticipatory	NA
		*n* = 24			24–82 years			
Ray et al. (2014)	UK	Current and former	MND Associations	NR	NR	92.3	Anticipatory	NA
	Australia	*n* = 13					Post-death	
Solomon and Hansen (2015)	US	Former	Participants known to principal investigator	25.0	NR	25.0	Post-death	NA
		*n* = 4			52–79 years			
Warrier et al. (2019)	India	Former	National tertiary care center	71.4	44.6 (9.3)	85.7	Post-death	NA
		*n* = 7			32–56 years			
Whitehead et al. (2012)	UK	Current and former	MND care and research center	50.0	NR	NR	Anticipatory	NA
		*n* = 28					Post-death	

*Note*. ^a^The study only reported gender distribution and age range for 24 informal carers originally recruited to the study. Of these, only 14 were included in the analysis. Current: carers currently caring for a person with MND; Former: carers who were caring for a person with MND in the past; ALS: amyotrophic lateral sclerosis; MND: motor neuron disease; PG-13: Prolonged Grief Disorder diagnostic tool; *SD:* standard deviations; NA: not applicable (not used); NR: not reported; UK: United Kingdom; US: United States.

### Selection process

Electronic search results were merged using an excel sheet. Duplicates were removed by one of the reviewers (APT). The initial screening of the titles and abstracts was conducted by APT. Full-text articles were assessed for eligibility by two reviewers independently (APT and TB). First, three qualitative studies and three quantitative studies were assessed for eligibility by APT and TB separately and compared to ensure accurate selection of the eligible studies. Following this, the remaining studies were evaluated for inclusion independently and disagreements were resolved through discussion.

### Data collection process

A purposely designed electronic data extraction sheet was used to extract the data. The form was first piloted by two reviewers (APT and NK) using two studies selected to represent different research methods. Following this, the data extraction sheet was piloted with another four studies by APT and TB to ensure accurate coding of information. Data were then extracted from all the remaining included studies independently by two reviewers (APT and TB). Discrepancies were resolved by consensus.

### Data item

The following information was extracted from each eligible study: the country where the research was conducted, type of carers (current or former), place of recruitment, sample size, mean and range of carer age, carers’ gender, carers’ relationship to care recipient, mean and range of duration of care, mean and range of period of bereavement for bereaved carers, and type of grief investigated (anticipatory, post-death, and/or prolonged). In addition, results related to factors associated (or not associated) with grief were extracted for quantitative studies. For qualitative studies, themes identified by original authors and findings related to factors linked with grief, such as quotations and text related to the findings, were extracted. If relevant information was not presented sufficiently, it was recorded as “not reported”. Original authors were not approached for clarification or further information.

### Study risk of bias assessment

The methodological quality and risk of bias in included studies were assessed using the Joanna Briggs Institute Critical Appraisal Tools. The Checklist for Analytical Cross-sectional Studies (Moola et al., 2017) was used for quantitative studies. This tool has eight items to assess different aspects of the methodological quality and reporting quality such as criteria for inclusion, description of subject and setting, identification and management of confounding factors and the validity of measures and statistical analysis used. The Checklist for Qualitative Research (Lockwood et al., 2015) was used for qualitative studies. This tool has 10 items to assess different aspects of the methodological quality and reporting quality such as the philosophical premises, data collection methods, interpretation of results, influence of the researcher and validity of the conclusion.

Two independent reviewers (APT and TB) assessed and scored each study using the checklists independently. Each checklist was first piloted with two studies to ensure both reviewers had the same understanding of the items. Following this, the reviewers scored all studies independently. Disagreements were resolved through discussion and a third reviewer (NK).

### Synthesis methods

The convergent integrated approach according to the Joanna Briggs Institute guidance on methodology for mixed methods systematic reviews was used to synthesize the findings (Stern et al., 2020). Following the guidance, quantitative data was converted into qualitized data and results from the quantitative studies and findings from the qualitative studies were thematically synthesized. For example, when extracting information regarding the results of statistical analysis conducted in quantitative studies, the relevant text summarizing the findings was recorded (e.g., ‘the factors that increased risk of PGD compared to low risk were having a recent bereavement and being a spouse or partner of the deceased'). Therefore, extracted information from each study included data in the forms of not only quotations but also text relevant to results and findings within each study (see supplementary Table 2 for detailed examples). First, APT and TB independently reviewed and created codes for extracted data. Initial codes were compared, discussed, and agreed. Codes were then reviewed by three reviewers (APT, TB, and NK). Similar codes were grouped into larger categories through discussion, which led to the development of meaningful interpretive themes and sub-themes. Relationships between themes and sub-themes were discussed and each theme and sub-theme were defined through discussion among three reviewers (APT, TB, and NK).

## Results

### Study selection

**Figure 1.**
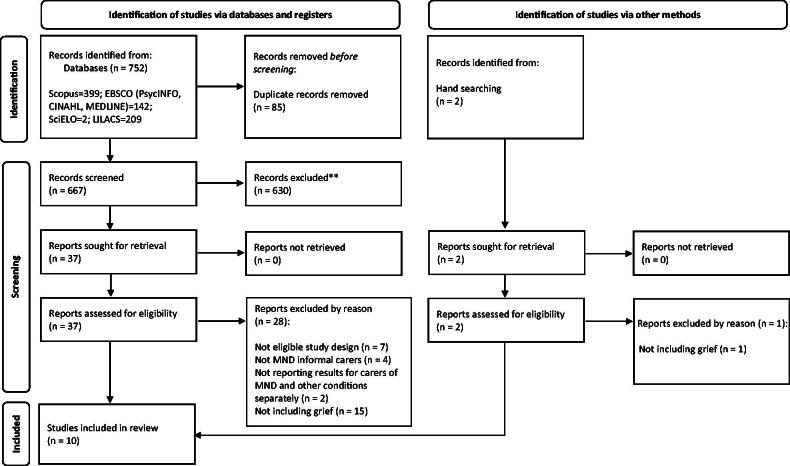
PRISMA 2020 flow diagram for included articles. *Note*. MND: motor neuron disease.

Figure 1 presents a flow diagram illustrating the study selection process. The search identified 752 studies, of which 85 were excluded as duplicates. The remaining 667 studies were screened based on title and abstract. Thirty-seven studies were deemed potentially relevant and therefore, full texts were obtained and subjected to full eligibility screening. This resulted in nine studies eligible for the review. Two additional studies were identified through a hand search, retrieved, and screened for inclusion, from which only one was eligible. In total, 10 studies were included in this review for analysis.

### Study and participant characteristics

#### Participant characteristics

The characteristics of the participants are summarized in Table 1. Of the 10 studies included, two studies were quantitative (cross-sectional) studies with the same sample (*n* = 393), though reported different outcomes. The remaining eight studies used a qualitative approach. These studies had a relatively small sample size ranging from 4 to 28 except for one study, which qualitatively analyzed free-text survey responses collected using the same sample as the two included quantitative studies (*n* = 393). Most studies were conducted in Australia (*n* = 6) and most studies recruited participants from MND Associations (*n* = 6). Most studies recruited former carers or both current and former carers, while only one study solely recruited carers currently supporting the person living with MND. Carer age ranged from 22 to 91 years across seven studies that provided the information. Most carers were women spouses of the person living with MND.

#### Outcome

The two quantitative studies included in the current review focused on post-death grief and PGD as outcomes and used the PGD diagnostic tool (PG-13) to measure prolonged grief responses. No quantitative study investigated factors associated with anticipatory grief. Of the eight qualitative studies included, one study solely targeted anticipatory grief by recruiting current carers and three studies focused on post-death grief only. The remaining four qualitative studies targeted experiences over a longer period and investigated both anticipatory and post-death grief or post-death and PGD.

### Risk of bias in studies

The results of methodological quality rating for the two quantitative studies are presented in the supplementary Table 3. One study presented a high methodological quality meeting all criteria. The other study demonstrated a lower quality due to variables of interest not being measured in a reliable way and confounding factors not being considered during the data analysis. The results of methodological quality rating for the eight qualitative studies are presented in the supplementary Table 4. The quality of included qualitative studies varied, with studies fulfilling between 4 and 9 out of 10 criteria of the tool. Most studies did not demonstrate a clear congruence between the philosophical perspective the study is based, and the methodological approach used. None of the studies acknowledged or addressed the influence of the researcher on the research or the research process on the researcher.

### Results of syntheses

**Figure 2.**
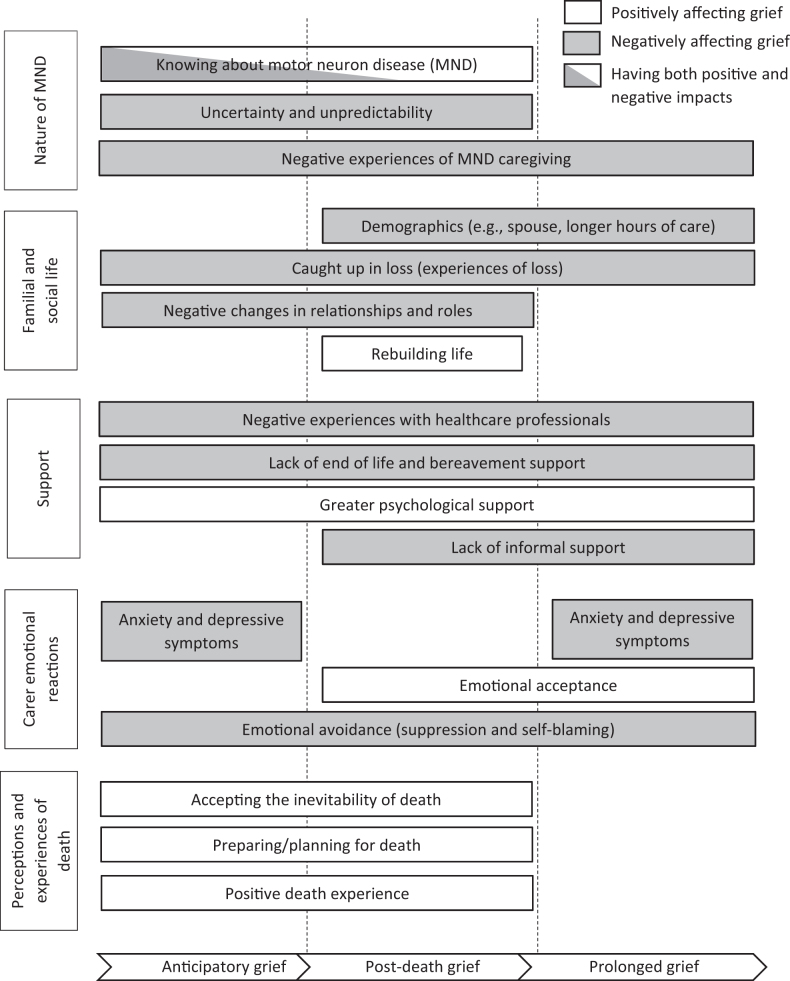
Factors affecting the different grieving processes. Rectangles show themes and sub-themes identified in the review.

Five overarching thematic categories were generated to illustrate factors associated with informal MND carers’ grief experiences. Figure 2 outlines the five key themes and sub-themes and how each sub-theme is associated with the different grieving processes (anticipatory, post-death and/or prolonged). The five overarching themes were (1) nature of MND, (2) familial and social life, (3) support, (4) carers’ emotional reactions and (5) perceptions and experiences of death. A list of illustrative quotations and summary text supporting each sub-theme is provided in supplementary materials (see supplementary Table 2).

#### Nature of MND

The findings demonstrated that the nature of MND, such as unclear progression of the condition and high demands of caregiving, was associated with the grieving processes of carers of people living with MND. Three sub-themes were identified: knowing about MND, uncertainty and unpredictability, and negative experiences of caregiving.

##### Knowing about MND

Two qualitative studies reported that knowing about the trajectory and progression of MND was linked to experiencing anticipatory and post-death grief (Aoun et al., 2012; Ray et al., 2014). For example, knowing the nature of MND in the early stage (incurable like other conditions such as cancer) brought greater feelings of hopelessness, having a negative impact on anticipatory grief (Aoun et al., 2012). Conversely, the findings demonstrated that the carers’ prior knowledge about MND can also have a positive impact on anticipatory and post-death grief processes (Ray et al., 2014). The carers’ prior knowledge enabled them to construct the death of the person living with MND as a positive release from MND, while carers’ lack of knowledge (being unprepared for the symptoms of dying) led to greater loss of hope, negatively affecting anticipatory and post-death grief processes (Ray et al., 2014).

##### Uncertainty and unpredictability

Three qualitative studies highlighted that the trajectory and progression of MND arose feelings of uncertainty and unpredictability in carers of people living with MND, impacting negatively on their anticipatory and post-death grief processes (Ray et al., 2014; Ray & Street, 2007; Warrier et al., 2019). The lack of a set trajectory in MND meant carers faced unforeseen changes, negatively affecting their anticipatory grief (Ray & Street, 2007). Additionally, the way the death of the person living with MND occurred, such as unexpected timing of death (Warrier et al., 2019), or it not following the usual course of MND (Ray et al., 2014), affected carers’ anticipatory and post-death grief negatively.

##### Negative experiences of MND caregiving

Four qualitative studies reported distress related to caregiving and re-experiencing such negative caregiving experiences had negative impacts on the three grieving processes (Aoun, Noonan, et al., 2021; Aoun et al., 2012; Poppe et al., 2022; Ray & Street, 2007). For example, carers’ exposure to a continual physical and emotional caregiving burden and the physical degeneration of the person living with MND had a negative effect on their anticipatory and post-death grieving processes (Poppe et al., 2022; Ray & Street, 2007). Moreover, symptoms of MND made the disease a difficult illness experience, and recalling such experiences after the death of the person living with MND (Aoun, Noonan, et al., 2021) and reexperiencing the painful emotions related to death (Aoun et al., 2012) were linked to PGD.

#### Familial and social life

Findings from the literature suggested that the demographic characteristics of the carer and the person living with MND, and the changes in their intimate relationship and social life impacted carers’ grieving processes. Four sub-themes were identified: demographics of the carer and the person with MND, caught up in loss, relationships and changes in roles, and rebuilding life.

##### Demographics (carer and person with MND)

Only one quantitative study exploring the association between carer- and patient-related demographic factors and PGD was identified (Aoun et al., 2020). Factors associated with a higher risk of PGD as measured by the PG-13 were: being a spouse or partner of the person living with MND, the age of the deceased person living with MND being less than 60 years, longer hours of care provided in the last 3 months prior to the death of the person living with MND, the time of caring being less than 1 year and a half and the recent death of the person living with MND (bereavement of less or equal to 12 months). Carers’ age, gender, employment status, cultural background, and education levels were not associated with higher PG-13 scores (Aoun et al., 2020). One qualitative study also identified that the long hours of caring negatively impacted carers’ post-death grief (Warrier et al., 2019).

##### Caught up in loss

Six of the eight qualitative studies reported carers’ loss of social life and purpose in life, and feelings of loneliness were associated with grieving processes (Aoun, Noonan, et al., 2021; Aoun et al., 2012; Poppe et al., 2022; Ray et al., 2014; Ray & Street, 2007; Warrier et al., 2019). The losses carers experienced during the trajectory of the disease, such as feeling deprived of a social life or retirement plans, were related to a negative anticipatory grief process (Ray & Street, 2007), while the loss of purpose in life experienced by carers after the person living with MND’s death was associated with post-death grief (Aoun, Noonan, et al., 2021). Carers also reported difficulty in mentally moving away from their caregiving role (Ray et al., 2014) and the person living with MND after their death (Aoun, Noonan, et al., 2021), which negatively impacted on both anticipatory and post-death grief. Loneliness due to the absence of the person living with MND (Poppe et al., 2022) and difficulties in adjusting to and reengaging in a new life without the person living with MND (Aoun, Noonan, et al., 2021) had a negative impact on post-death grief. Moreover, a constant series of loss experiences leading to feelings of hopelessness were negatively associated with PGD (Aoun et al., 2012).

##### Relationships and changes in roles

Carers’ perceptions of the relationship between them and the person living with MND and the changes in roles in their relationship were found to be important factors affecting carers’ grieving processes in four qualitative studies (Aoun et al., 2012; Ray et al., 2014; Ray & Street, 2007; Solomon & Hansen, 2015). For example, feeling uncertain in terms of what it meant to be a partner (Ray & Street, 2007) and experiencing a change in roles from being a spouse to a carer (Aoun et al., 2012) were predominant factors negatively affecting anticipatory grief. Being able to focus on the relationship with the person living with MND in a safe and relaxed context (i.e., home) at the end of life impacted positively on carers’ anticipated and post-death grief processes (Solomon & Hansen, 2015). An open communication style used among family members also created a rich environment towards the end of life and emphasis on family relationships, which was related to a positive post-death grief process (Solomon & Hansen, 2015).

##### Rebuilding life

Several factors impacting positively on carers’ post-death grief were identified in two qualitative studies. These included factors related to how carers re-engaged in a new life and kept active after the person living with MND’s death (Aoun, Noonan, et al., 2021; Poppe et al., 2022). For example, spending time on self-care, self-reflection, and self-improvement helped carers to cope with post-death grief (Aoun, Noonan, et al., 2021) Furthermore, doing new things (Aoun, Noonan, et al., 2021), keeping busy (Aoun, Noonan, et al., 2021; Poppe et al., 2022), and being with others (Poppe et al., 2022) were also important factors in effectively managing their grieving process after the death of the person living with MND.

#### Support

The quality of support provided to MND informal carers and how it was received by them, during and after the trajectory of the disease, was presented as a fundamental aspect impacting carers’ grief. Four sub-themes were identified: negative experiences with healthcare professionals, end of life and bereavement support, psychological support, and informal support.

##### Negative experiences with healthcare professionals

Three qualitative studies found that the undesirable interactions with healthcare professionals experienced by carers negatively affected their grieving processes (Aoun, Noonan, et al., 2021; Aoun et al., 2012; Ray et al., 2014). For example, healthcare professionals’ unempathetic way of communicating the diagnosis of MND had a negative impact on anticipatory grief (Aoun et al., 2012). Carers’ disrespected desires concerning healthcare practices, such as resuscitation decisions, and the lack of choices over the healthcare provided negatively affected carers’ anticipatory and post-death grief (Ray et al., 2014). Furthermore, the failure of support from formal services also negatively impacted post-death grief, leaving carers with feelings of anger which was associated with risk of prolonged grief (Aoun, Noonan, et al., 2021).

##### End of life and bereavement support

Four qualitative studies (Aoun, Noonan, et al., 2021; Aoun et al., 2012; Ray et al., 2014; Whitehead et al., 2012) and one quantitative study (Aoun et al., 2020) identified that end of life and bereavement support received by carers affected their grieving processes. For example, satisfactory and favorable experiences with hospital staff at the person living with MND’s end-of-life (Ray et al., 2014) affected anticipatory and post-death grief processes positively. Bereavement support received after the person living with MND’s death also positively affected post-death grief (Whitehead et al., 2012), while not having access to support or receiving unfavorable support in the early post-death days and the withdrawal of services and the end of contact from services negatively affected carers’ post-death bereavement (Whitehead et al., 2012) and heightened the risk of PGD (Aoun et al., 2020). The lack of the person living with MND’s early access to palliative care services and home-based services were negatively related to carers’ PGD (Aoun et al., 2012).

##### Psychological support

Three qualitative studies (Aoun, Noonan, et al., 2021; Poppe et al., 2022; Warrier et al., 2019) and one quantitative study (Aoun, Cafarella et al., 2021) identified that psychological and counseling services received by carers had positive impacts on grieving processes. Counseling received while providing care and after the death of the person living with MND was positively associated with carers’ both anticipatory and post-death grief (Aoun, Noonan, et al., 2021). Psychological/emotional support was found to have a positive impact on post-death grief in the qualitative studies (Poppe et al., 2022; Warrier et al., 2019), and not receiving such support was associated with risk of PGD in the quantitative study (Aoun, Cafarella et al., 2021).

##### Informal support

Two qualitative studies (Aoun, Noonan, et al., 2021; Poppe et al., 2022) and two quantitative studies (Aoun, Cafarella et al., 2021; Aoun et al., 2020) identified that informal support received by carers influenced their grieving processes. Receiving support from MND specific support organizations, local MND Associations, friends, community, and family members (Aoun, Noonan, et al., 2021) as well as support from peers (Poppe et al., 2022) had a positive effect on carers’ post-death grief. Lack of support from family and MND-related organizations (Aoun, Cafarella et al., 2021) as well as conflictual family functioning (Aoun et al., 2020) were found to be associated with a higher risk of PGD in quantitative studies (Aoun, Cafarella et al., 2021; Aoun et al., 2020).

#### Carers’ emotional reactions

Carers’ psychological symptoms and how carers responded to such psychological challenges were associated with carers’ grieving processes. Three sub-themes were identified: anxiety and depressive symptoms, emotional acceptance, and emotional avoidance.

##### Anxiety and depressive symptoms

One qualitative study (Whitehead et al., 2012) and one quantitative study (Aoun et al., 2020) identified that anxiety and depressive symptoms were negatively linked to carers’ grieving processes. Worries about the future such as carers worrying about how they would cope as the disease advanced, how death would occur and whether the child/ren of a person living with MND would cope after the loss of a parent were found to be linked to carers’ anticipatory grief in the qualitative study (Whitehead et al., 2012). Higher anxiety and depressive symptoms assessed by standardized measures were found to have a strong association with higher PGD risk in the quantitative study (Aoun et al., 2020).

##### Emotional acceptance

Three qualitative studies identified that carers’ ability to accept the illness, the recognition they had provided good care and their capability of embracing the feelings of grief had positive impacts on their grieving processes (Aoun, Noonan, et al., 2021; Aoun et al., 2012; Poppe et al., 2022). Accepting the bereavement experience as part of life, expressing and allowing grief as part of adapting to the loss of the person living with MND (Aoun, Noonan, et al., 2021), and viewing grief as a natural process (Poppe et al., 2022) were associated with a positive post-death grieving process. Comments from others reassuring carers that they had done a good caring job, also had positive impacts on their post-death grief (Poppe et al., 2022). Carers’ ability to accept the person living with MND’s illness as terminal (Aoun et al., 2012), their recognition that they did not miss what the illness encountered, and their capability of appreciating the days they had together with the person living with MND (Aoun, Noonan, et al., 2021) were associated with a lower risk of developing PGD.

##### Emotional avoidance

Five qualitative studies identified that carers’ tendency to avoid or control thoughts and feelings related to caregiving and their judgmental attitudes, such as self-blaming and self-doubting, had negative impacts on the three grieving processes (Aoun, Noonan, et al., 2021; Aoun et al., 2012; Ray & Street, 2007; Warrier et al., 2019; Whitehead et al., 2012). Self-doubting, such as carers questioning themselves regarding their capacity to continue with caregiving demands had a negative impact on their anticipatory grief process (Ray & Street, 2007). Self-criticism and guilt, such as carers thinking that they could have provided better care were associated with carers’ post-death grief (Warrier et al., 2019; Whitehead et al., 2012). Trying or pretending to remain strong after the person living with MND died (Aoun, Noonan, et al., 2021; Warrier et al., 2019) also had negative impacts on carers’ post-death grief. Limiting emotional reactions to the person living with MND’s illness and avoiding thinking about the person living with MND’s death were linked to a higher risk of PGD (Aoun et al., 2012).

#### Perceptions and experiences of death

Anticipating and preparing for death and reactions to how the death occurred were associated with carers’ grieving processes. Three sub-themes were identified: accepting the inevitability of death, preparing/planning for death, and death experience.

##### Accepting the inevitability of death

This sub-theme was evidenced by two qualitative studies (Ray et al., 2014; Warrier et al., 2019). Carers’ acceptance of the inevitable death as the end of suffering and no further losses for the person living with MND positively affected their anticipatory and post-death grief (Ray et al., 2014). Additionally, carers experienced a sense of relief when the person living with MND died as the suffering ended, leading to a positive post-death grief process (Warrier et al., 2019).

##### Preparing/planning for death

Four qualitative studies reported that the carer and the person living with MND planning for death together, such as discussing wishes about how they preferred death to occur, helped carers prepare for their grieving processes before death and during their bereavement (Aoun, Noonan, et al., 2021; Poppe et al., 2022; Ray et al., 2014; Solomon & Hansen, 2015). For example, having conversations and making plans on dying and death allowed carers to experience some comfort and these had positive impacts on carers’ anticipatory and post-death grief (Ray et al., 2014) and lowered the risk of developing PGD (Aoun, Noonan, et al., 2021). Furthermore, supporting the person living with MND in their desire to die at home, created an ideal environment for carers’ post-death grieving process and gave carers time to be prepared mentally and emotionally for the person living with MND’s death (Solomon & Hansen, 2015).

##### Death experience

Four qualitative studies identified the importance of carers’ experiences on how the death occurred and their reactions towards it and how these affected their grieving processes (Poppe et al., 2022; Ray et al., 2014; Solomon & Hansen, 2015; Whitehead et al., 2012). The person with MND dying at home was found to have a positive effect on anticipatory grief (Solomon & Hansen, 2015) and on post-death grief (Ray et al., 2014; Solomon & Hansen, 2015) as it enabled time to say goodbye to the person living with MND. Most of the factors related to this sub-theme (death experience) impacted positively on post-death grief. These experiences included seeing that death occurred before MND had become too severe (Whitehead et al., 2012), perceiving death as a non-traumatic event (Poppe et al., 2022), carers feeling that they had closure with the person living with MND (Whitehead et al., 2012), and feeling that the person living with MND had no unaccomplished desires or expectations and was not afraid of dying (Solomon & Hansen, 2015). However, when the death of the person living with MND was felt to be undignified, carers were left with regrets about how death had occurred, leading to negative grieving processes (Ray et al., 2014).

## Discussion

This review aimed to identify factors associated or linked with anticipatory grief, post-death grief, and PGD in family carers of people living with MND. The findings suggested that there may be factors particularly important to target during the early diagnosis of MND as they might affect both anticipatory and post-death grief. These factors included carers’ need for greater knowledge about the progression of the disease, coping with changes in the relationship with the care recipient, reduction of anxiety and depressive symptoms of carers, and encouragement of planning for the death of the person living with MND. Findings also highlighted that how carers adjusted and rebuilt life without the person living with MND such as not being alone and engaging in activities, are important factors to consider during their post-death period. There were factors that were more likely to be relevant to the risk of developing PGD, such as being a spousal carer, formal and informal support received, and emotional acceptance. Factors that may affect all three types of grief were also identified, such as negative experiences of caregiving, getting caught up in loss experiences, lack of end of life and bereavement support, lack of psychological support and emotional avoidance coping.

Some factors identified in this review were consistent with the findings of previous reviews on factors associated with grief in other populations such as dementia, patients in intensive care units and the general bereaved population (Chan et al., 2013; Crawley et al., 2022; Mason et al., 2020; Sanderson et al., 2022) such as carer depression (Chan et al., 2013; Crawley et al., 2022; Mason et al., 2020), carer anxiety (Mason et al., 2020), lack of social support (Crawley et al., 2022; Mason et al., 2020), being in a spousal relationship (Chan et al., 2013; Crawley et al., 2022; Mason et al., 2020; Sanderson et al., 2022), difficulty in accepting the death (Mason et al., 2020), being unprepared for death (Chan et al., 2013; Crawley et al., 2022; Sanderson et al., 2022), and not accepting the death (Mason et al., 2020). This consistency in factors identified may be because, similar to people living with MND, people living with other chronic conditions, such as dementia, are often cared by their spouses (Brodaty & Donkin, 2009; Johansson et al., 2021), and caregiving can affect informal carers’ mental health regardless of the condition (Savage & Bailey, 2004; Schulz & Sherwood, 2008). Moreover, it has been reported that 50% of people living with MND will present with behavioral changes and 15% will develop frontotemporal dementia (Bäumer et al., 2014). This overlap between two neurodegenerative diseases (i.e., MND and dementia) may explain why carers of both populations face similar grieving experiences.

There are no systematic reviews on factors affecting grief in carers of people living with other rapidly progressing neurodegenerative conditions such as Parkinson’s disease, or other neurological conditions such as traumatic brain injury and stroke. Therefore, it is not possible to compare the findings of this systematic review with those of carers of people affected by neurodegenerative conditions of fast progression and poor clinical prognosis. However, there is some emerging evidence. For example, a qualitative study involving five carers of people who had suffered a stroke identified similar factors affecting their grief experiences such as changes in roles and relationships (Hughes & Cummings, 2020). Two further qualitative studies, which involved 16-24 carers of people who had suffered a stroke, identified similar factors such as uncertainty about the future and negative emotions generating emotional distress (Gosman-Hedström & Dahlin-Ivanoff, 2012; McCurley et al., 2019). One quantitative study involving 29 carers of people living with Parkinson’s disease found a strong relationship between depression, burden and anticipatory grief (Fox et al., 2020). Due to the limited evidence in other carer populations, it is not possible to conclude whether the factors identified in this systematic review are relevant to wider carer populations.

Nevertheless, it is important to highlight that some of the factors found in the current review may be particularly pertinent for this population such as the knowledge of MND, the uncertainty and unpredictability of the disorder and lack of emotional acceptance and presence of emotional avoidance. This might be due to the lack of a set trajectory in MND and the rapid progression of MND symptoms (Bäumer et al., 2014). These findings suggest that tailored carer interventions for this population may be useful to help carers cope with their grieving processes prior to and after the death of the person living with MND and examining these potential interventions is an important next step.

### Limitations of evidence

The number of included studies was relatively small (n = 10) and were mostly from Australia, and thus the results may not be generalizable to populations from other countries and cultures. Most included studies were qualitative studies with relatively small datasets, meaning the generalization of the findings might be limited. Furthermore, all factors were not consistently explored across the three grieving processes. For example, demographics were mainly explored in relation to PGD in a single quantitative study. Therefore, the impact of such factor on anticipatory grief is unknown. These suggest that the findings of this review need to be interpreted with caution.

### Implications

#### Clinical implications

A recent systematic review on interventions targeting psychological well-being for carers of people living with MND (Cafarella et al., 2022), which included a single study that targeted grief among other outcomes, concluded that the majority of studies did not demonstrate significant benefits on outcomes such as carer anxiety and depression. Interventions used in the studies included in the review were mainly single-component interventions (e.g., mindfulness alone, dignity therapy). Considering the findings of the current review, multi-component interventions may be more beneficial for treating grief in this population.

Early educational interventions aimed at providing information about MND symptoms, including how the disease may progress differently across people living with MND, and available support resources could be beneficial for coping with both anticipatory and post-death grief. However, these should be provided with psychological support or counseling as increased knowledge could lead to greater anticipatory grief before it can help carers to cope with post-death grief. Psychological interventions and counseling could also be beneficial to support carers from the point of diagnosis. These could help carers to cope with the emotional impact of the changes in their roles and relationships, reduce anxiety and depressive symptoms and facilitate emotional acceptance rather than emotional avoidance. Designing interventions tailoring end-of-life care for planning for the death could reduce post-death grief symptoms and PGD risk. Additionally, it would be beneficial to consider educational interventions with healthcare professionals to raise awareness and provide information on how the relationship between healthcare professionals and carers impacts carers’ emotional wellbeing.

#### Research implications

Factors associated with anticipatory grief were less reported and no quantitative study explored factors associated with this grieving process. This might be due to the lack of a standardized measure to assess anticipatory grief in this population. Future research should consider designing a validated tool to screen anticipatory grief in this population, which allows researchers to identify factors associated with grieving experiences prior to death. Longitudinal studies assessing the impact of factors affecting anticipatory and post-death grief overtime should be conducted to build on further understanding of the strongest predictors of grief in this population. Moreover, future research should explore the efficacy of interventions targeting the factors identified in this review on the grieving processes in carers of people living with MND.

The aforementioned clinical recommendations could potentially be generalizable to other progressive disorders. Future research could explore whether similar risk factors equally contribute to grieving reactions across different carer populations. This would contribute to the general knowledge of how to address grieving experiences in informal carers.

### Methodological limitations

Searches were limited to studies published in English, Spanish, and Portuguese in this review, which may have introduced bias. No studies were excluded based on quality in this review and some of the included qualitative studies demonstrated relatively low quality. Due to identifying limited quantitative studies, both quantitative and qualitative results were incorporated into the same thematic synthesis and the term association was used to demonstrate factors such as situations or conditions that may accompany the grieving processes throughout this review. Since most data for the synthesis was qualitative, we can only suggest factors that the original participants or authors linked to, or mentioned in relation to grief processes and cannot verify statistically whether these factors are associated with grief.

## Conclusion

Despite the limitations, this review demonstrated that various factors could affect anticipatory, post-death, and PGD in informal carers of people living with MND. Some of these factors may be particularly pertinent to this population, such as the knowledge of MND (as a relative rare condition and its progression), the uncertainty and unpredictability of the progression of the disorder, and emotional acceptance and avoidance. Therefore, tailored interventions targeting specific factors affecting grieving processes in this population might be useful. Cross-sectional studies using a large sample size with a particular focus on factors associated with anticipatory grief and longitudinal studies exploring factors affecting grieving processes (anticipatory, post-death, and PGD) could further elucidate the role of specific clinical and social factors in the various types of grief in MND.

## Supplementary Material

Supplemental Material
