# Supplementary material for: Factors associated with grief in informal carers of people living with Motor Neuron Disease: A mixed methods systematic review
Source: Death Stud. 2023 Mar 30;48(2):103–17. doi: 10.1080/07481187.2023.2191351 (PMC11601043; doi:10.1080/07481187.2023.2191351)
Supplement: Supplemental Material [file UDST_A_2191351_SM2158.docx]

| **Supplementary Table 1**  *Search strategy terms* | | | | |
| --- | --- | --- | --- | --- |
| **Database** | | **Search terms** | **Search filter** | |
| PsycINFO, CINAHL, MEDLINE | (carer* OR caregiver* OR spous* OR partner* OR child* OR parent* OR famil*)  AND  (“amyotrophic lateral sclerosis” OR “motor neuron disease” OR “motor neurone disease” OR “Lou Gehrig's disease” OR “MND” OR “ALS” OR “neurodegenerative disease” OR “neurodegenerative disorder”)  AND  (grief OR bereavement OR bereaved OR mourning OR griev*)  AND NOT  (“systematic review”) | | | Any field |
| SCOPUS | (carer* OR caregiver* OR spous* OR partner* OR child* OR parent* OR famil*)  AND  (“amyotrophic lateral sclerosis” OR “motor neuron disease” OR “motor neurone disease” OR “Lou Gehrig's disease” OR “MND” OR “ALS” OR “neurodegenerative disease” OR “neurodegenerative disorder”)  AND  (grief OR bereavement OR bereaved OR mourning OR griev*)  AND NOT  (“systematic review”) | | | Limited to the article title, abstract and keywords |
| LILACS | (cuidador OR pareja OR espos* OR hij* OR madre* OR padre* OR pai* OR mae* OR familia* OR casal OR filhos OR pat* OR mat* OR marido)  AND  ("esclerosis lateral amiotrofica" OR "enfermedad de la motoneurona" OR "enfermedad de la neurona motora" OR "enfermedad de Lou Gehrig" OR "ELA" OR "enfermedad neurodegenerativa" OR "desorden neurodegenerativo" OR “esclerose lateral amiotrofica” OR “doença do motoneuronio” OR “doença do neuronio motor” OR “doença neuro degenerativa” OR “desordem neuro degenerativa”)  AND  (duel* OR perdida OR afliccion OR luto OR perda OR afliçao)  AND NOT  (“revisão sistemática” OR “estudio sistematico) | | | Any field |
| SciELO | cuidador OR pareja OR espos* OR hij* OR madre* OR padre* OR pai* OR mae* OR familia* OR casal OR filhos OR pat* OR mat* OR marido)  AND  ("esclerosis lateral amiotrofica" OR "enfermedad de la motoneurona" OR "enfermedad de la neurona motora" OR "enfermedad de Lou Gehrig" OR "ELA" OR "enfermedad neurodegenerativa" OR "desorden neurodegenerativo" OR “esclerose lateral amiotrofica” OR “doença do motoneuronio” OR “doença do neuronio motor” OR “doença neuro degenerativa” OR “desordem neuro degenerativa”)  AND  (duel* OR perdida OR afliccion OR luto OR perda OR afliçao)  AND NOT  (“revisão sistemática” OR “estudio sistematico) | | | Any field |

| **Supplementary Table 2**  *Illustrative Quotations* | | |
| --- | --- | --- |
| **Theme and sub-theme** | **Illustrative quotations (examples) from qualitative studies and**  **key findings from quantitative studies** | **Record reporting sub-theme** |
| **Nature of MND** |  |  |
| Knowing about MND | Feelings of hopelessness were reported by 11 carers, including all who met the criteria for prolonged grief. The hopelessness was described by one participant as ‘the most defeating thing’. The sense of hopelessness that participants felt seemed to be compounded further when they contrasted MND to cancer. For example, one stated, “With cancer there is hope; with MND, there is nothing”. (Aoun et al., 2012)  Although in one case, finding the person dead was a surprise, the trajectory to date and the caregiver’s knowledge of MND enabled her to construct death as a positive event. “I just went into [name] room and there he was...the expression on his face indicated to me that he was peaceful, he stayed at home with us for 24 hours”. (Ray et al., 2014) | Aoun et al., 2012; Ray et al. 2014. |
| Uncertainty and unpredictability | People living with MND reported living in a constant state of uncertainty as they confronted the ongoing losses. They expressed frustration that, unlike cancer, this disease had no set trajectory and that they must manage constant changes without knowing exactly what these changes might be: “With a disease like this no-one can give you its parameters or where it’s going or how long it’s going…This is so undetermined you just don’t know it from one moment to the other”. (Ray & Street, 2007)  The unexpected nature of [x] partner’s death, while in hospital, was contrary to her understanding of the illness trajectory (she imagined him becoming bedbound and needing increasing care) and deprived her of being at his side in the death event. “I wasn’t there with him, he died alone, the end was quick, unexpected, it shocked me, I was unprepared. He wasn’t going to die; it wasn’t part of the plan”. (Ray et al., 2014) | Ray & Street, 2007; Ray et al., 2014; Warrier et al., 2019. |
| Negative experiences of MND caregiving | Participants in every risk [of complicated grief] category reported experiencing a range of emotions such as anger, depression, regret, guilt, sadness, and shock as part of their grief…Caregivers at moderate risk [of complicated grief] were more often distressed by remembering the MND symptoms: “I find it very difficult to put the images of my husband’s body wasting away before my eyes out of my head” and “I couldn’t bear having anything that reminded me of MND”. (Aoun, Noonan et al., 2021)  Several caregivers expressed that they felt severe burden or grief after bereavement. They noted how bereavement after death from ALS was different due to the extensive nature of caregiving provided to the person with ALS. A widower who had cared for his wife with ALS at home succinctly described the bereavement as different from other deaths in light of the exhausting and long caregiving needed for his wife. “It is not like that/I have already experienced several deaths: parents, brothers and everything else … That was much less burdening, but if you have it in a way where you are still exhausted, where the reserves decrease and then comes the case X. …[ehm] I was amazed how much energy you get when you need it. Because I don’t know where you get it from”. (Poppe et al., 2022) | Aoun et al., 2012; Aoun, Noonan et al., 2021; Poppe et al., 2022; Ray & Street, 2007. |
| **Familial and social life** |  |  |
| Demographics (person living with MND and carer) | A quantitative study demonstrated that marital status, education level, relationship to the deceased, the period of bereavement, length of care, type of involvement in caring, and intensity of caring in hours significantly differed between the three risk groups (high, moderate, and low risks of PGD) (all *p* < 0.05). After adjusting for confounding variables, the factors that increased risk of PGD compared to low risk were having a recent bereavement and being a spouse or partner of the deceased. (Aoun et al., 2020)  A quantitative study demonstrated that age, gender, employment status, and cultural background of the bereaved did not differ significantly between the three risk groups (all *p* > 0.05). (Aoun et al., 2020) | Aoun et al., 2020; Warrier et al., 2019. |
| Caught up in loss | [One] caregiver talked about wrestling with the images of what might have been, especially the retirement that they had planned “You just keep in grieving for the life you have lost, for the life you were going to have, the life you are not going to have (cries) and for all he has lost. It’s awful”. (Ray & Street, 2007)  Being alone and struggling with loneliness was a common experience for people in the moderate and high [grief risk] categories. Caregivers reported “it being difficult suddenly living alone” and a feeling of “loss of purpose...nothing prepares you for being alone”. (Aoun, Noonan et al., 2021) | Aoun et al., 2012; Aoun, Noonan et al., 2021; Poppe et al., 2022; Ray & Street, 2007; Ray et al., 2014; Warrier et al., 2019. |
| Relationships and changes in roles | Most participants described the loss of what is means to be a partner and loss of what the patient gave in their relationship…Commitment to her relationship with her partner was providing some sustenance for one caregiver; however, the impact of the physical and emotional responsibility and the loss of a reciprocal relationship increased caregiver strain and substantially changed the relationship: “I don’t feel like I have a better half, I feel like I’ve got a baby. So, yeah, but I mean, I still love him to death and I’d do anything for him, but, you’ve sort of been left with the whole burden on you, to carry. I feel like I’ve got a baby, I haven’t got a husband”. (Ray & Street, 2007)  “She didn’t express any fear of dying. She gave me plenty of time to be prepared mentally and emotionally. And she and I were able to talk, to experience each other. And I was able to experience my love for her. So I didn’t have any unfulfilled desires that interfered with or contaminated my bereavement. There was complete closure. I was ready when she was ready”. (Solomon & Hansen, 2015) | Aoun et al., 2012; Ray & Street, 2007; Ray et al., 2014; Solomon & Hansen, 2015. |
| Rebuilding life | “Doing new things” and “keeping busy” were the most common ways family caregivers coped with their loss in the low- and moderate-risk [grief] categories. Caregivers described actively needing to re-engage with their life. “I re-joined the bowling club”; “I just did an amazing overseas trip” and “I went on a three day walk, and it was better than any drugs the doctors gave me”. (Aoun, Noonan et al., 2021)  The next important finding in positive bereavement experiences is related to self-reflection and self-improvement. Once again, caregivers who were in the low-risk category are most represented in this section. Caregivers expressed feelings of gratitude, empowerment and positive emotions: “Gratitude for my life and what I have/ Yes I now realise more than ever the shortness of life and need to live for now, in the present be more mindful and patient and understanding of others and self. I feel more able to make my own decisions –empowered and feel I am now on a reflective learning journey. It’s the quality of the journey NOT the end result”. (Aoun, Noonan et al., 2021) | Aoun, Noonan et al., 2021; Poppe et al., 2022. |
| **Support** |  |  |
| Negative experiences with healthcare professionals | The lack of empathy from medical professionals left the participants feeling shocked, bewildered, angry and devastated. One participant recalled: “The neurologist told us that he knew someone with MND and that they shot themselves right away. Hearing that was just terrible “cause here I am thinking we will beat this. Nah, we didn’t go to that neurologist again”. (Aoun et al., 2012)  Breaches of the patient wishes and minimal support from health-care workers were reported…The “not for resuscitation” order and the family caregiver’s wishes were ignored. The family caregiver felt powerless to change the situation. The ensuing chaos left the caregiver with regrets about the undignified nature of his wife’s death. “I couldn’t [make them stop], stop it, don’t give her artificial resuscitation but…”; “Well she’d already started, you know, but those carers were in a hell of state and hysterical, well one of them was hysterical … a green no resuscitation and that ought to be in big letters and told to all the carers. That’s the one disappointment; I would have loved her to go peacefully”. (Ray et al., 2014) | Aoun et al., 2012; Aoun, Noonan et al., 2021; Ray et al., 2014. |
| End of life and bereavement support | Not being provided with support in the early days following her husband’s death was exceptionally distressing for one bereaved carer. She stressed the importance of a sense of closure following the death, particularly because the withdrawal of services and ending of contacts came across as a stark administrative process “When (patient) died (Specialist nurse) never got in touch with me [. . .] I was absolutely devastated about that, I couldn’t get over it, couldn’t get over it. . .You are just cut off then [after the death], it goes in a drawer, the notes go in a drawer”. (Whitehead et al., 2012)  Three of the five participants who demonstrated prolonged grief had accessed palliative care services less than one month before the death of their spouse. In addition, four out of the five participants who had prolonged grief and had used palliative care services reported gaining access to homebased services later than they would have liked. (Aoun et al., 2012) | Aoun et al., 2012; Aoun et al., 2020; Aoun, Cafarella, et al., 2021; Aoun, Noonan et al., 2021; Poppe et al., 2022; Whitehead et al., 2012; Ray et al., 2014. |
| Psychological support | Those people at moderate and high [grief] risk were more likely to recommend professional support “Get as much counselling throughout the caring period and after for at least six months”. (Aoun, Noonan et al., 2021)  In general, family caregivers mentioned support needs for bereaved children as “the children themselves grieved, each in their own way”. A few participants also stated that support for children came mostly from the family or schools. These family caregivers mentioned that psychological support for children would have been helpful but was not available. Participating caregivers also mentioned psychological growth on their and their children’s part. (Poppe et al., 2022) | Aoun, Cafarella, et al., 2021; Aoun, Noonan et al., 2021; Poppe et al., 2022; Warrier et al., 2019. |
| Informal support | The strong theme across all the [grief risk] categories was that social and family support was essential. Caregivers in the low-risk category frequently provided statements such as: “One gets on with life and keeps in touch with family, friends and hobbies” and “I was very fortunate to have the friend and family I have. They have been with me whenever I needed support, distraction or someone to reflect with”. (Aoun, Noonan et al., 2021)  A quantitative study revealed that after adjusting for confounding variables, the factor that increased risk of PGD compared to low risk was poor family function (low involvement or conflictual family function) (*p* < 0.05). (Aoun et al., 2020) | Aoun et al., 2020; Aoun, Cafarella, et al., 2021; Aoun, Noonan et al., 2021; Poppe et al., 2022. |
| **Carer emotional reactions** |  |  |
| Anxiety and depressive symptoms | “I mean I know there has been a vast deterioration in the last three months and I keep thinking what’s it going to be like in the next three months and the three months after that, I don’t know how I will cope, I will cope but I don’t know how I will cope, and I am worried”. (Whitehead et al., 2012)  A quantitative study demonstrated prolonged grief was significantly associated with anxiety and depression. After adjusting for confounding variables, when comparing high-risk PGD group vs low-grief PGD group, anxiety (*p* = 0.015) and depression (*p* < 0.001) were found to be factors that increased risk of PGD. Depression was found to increase the risk of PGD when comparing high-risk group vs moderate-risk group (*p* = 0.008) and moderate-risk group vs low-risk group (*p* = 0.011). (Aoun et al., 2020) | Aoun et al., 2020; Whitehead et al., 2012. |
| Emotional acceptance | Those participants who did not meet the criteria for prolonged grief generally indicated in their interviews that they accepted the notion of their partner dying, right from their diagnosis. For instance, one stated, “From the start, definitely, yes. Had no problem with that in the sense of accepting, you know, we’d had a really good life, we were both in our sixties”. (Aoun et al., 2012)  It was common for caregivers in the low-risk category to view expressing and allowing grief as a critical part of adapting, suggesting “The grieving process to wash its way through. Don’t ignore it. Don’t rush it and have tools/people on hand for when it gets too much” and “Stay open. Allow yourself to grieve. Recognise that not all days are going to be bad”. (Aoun, Noonan et al., 2021) | Aoun et al., 2012; Aoun, Noonan et al., 2021; Poppe et al. 2022. |
| Emotional avoidance | The participants who met the criteria for prolonged grief minimised their emotional reactions to their partner’s illness by ‘switching off’ their emotions in order to manage the day-to-day responsibilities of care. One participant described becoming “robotic and mechanical”, while another described trying to “keep busy, not think about it, avoid any discussion, avoid any acknowledgment [and] just keep going”. (Aoun et al., 2012)  Four of the six participants who met the criteria for prolonged grief avoided thinking about and re-experiencing the painful emotions associated with the death of their partner. For instance, one described having “a complete shutdown” and another stated, “There are things I’ve left undone because I haven’t been able to face them”. (Aoun et al, 2012) | Aoun et al., 2012; Aoun, Noonan et al., 2021; Ray & Street, 2007; Warrier et al., 2019; Whitehead et al., 2012 |
| **Perceptions and experiences of death** |  |  |
| Accepting the inevitability for death | Caregivers were able to construct death as the final part of the MND disease process. Despite having a variety of experiences of the dying process, caregivers reflected that while their loss of their partner was extremely significant; “there is a huge space there that [person] occupied”; they did not want them to continue to suffer with MND or go on facing the continual losses. “I wouldn’t have liked her to get any worse…the body was not worth having, I wouldn’t have wished that [MND] on anyone”. (Ray et al., 2014)  Many caregivers expressed the relief that the suffering for the individual has come to an end. “For eighteen days after discharge, we really struggled… But not more than that, and I am happy about that”. (Warrier et al., 2019) | Ray et al., 2014; Warrier et al., 2019. |
| Preparing/planning for death | “She gave me plenty of time to be prepared mentally and emotionally. And she and I were able to talk, to experience each other. And I was able to experience my love for her. So I didn’t have any unfulfilled desires that interfered with or contaminated my bereavement. There was complete closure. I was ready when she was ready”. (Solomon & Hansen, 2015)  In cases where conversations about dying and death had occurred, positive experiences were reported. Plans had been made, and caregivers were able to achieve some sense of comfort. “He told us everything he wanted; he always had done from the end of last year. He said you know I want this, I don’t want that”. (Ray et al., 2014) | Aoun, Noonan et al., 2021; Poppe et al., 2022; Ray et al., 2014; Solomon & Hansen, 2015. |
| Death experience | All three of the patient’s adult children, and the patient’s spouse, spoke eloquently about how being situated at home, supporting the patient in her wish to die there, allowed a safe and relaxed context to focus on relationships. They suggested this not only helped the patient feel her life was fulfilled, but also offered an ideal environment for their own anticipatory loss and bereavement: “Because she was comfortable and because we were doing everything she wanted, it made it so much easier for us. I don’t think we have regrets. I don’t think we feel guilty about needless procedures that traumatized her or us”. (Solomon & Hansen, 2015)  Generally, pointing to the normality of grief for most caregivers, one daughter of a person with ALS described not needing to access psychological support due to the non-traumatic nature of death from ALS in her case: “Neither of us did. But I think simply because it/it was somehow good and it wasn’t a traumatic experience for us actually/it was bad, sure, but it wasn’t [ehm]/I imagined it worse”. (Poppe et al., 2022) | Poppe et al., 2021; Ray et al., 2014; Solomon & Hansen, 2015. |

*Note*. MND = motor neuron disease. ALS = amyotrophic lateral sclerosis. PGD = Prolonged Grief Disorder.

**Supplementary Table 3**

*Quality rating of quantitative studies included in the review (Joanna Briggs Institute appraisal checklist for analytical cross-sectional studies)*

| Author(s) (year) | Item 1  Criteria for inclusion | Item 2  Description of subject/setting | Item 3  Measurement of the exposure | Item 4  Measurement of the condition | Item 5  Identification of confounding factors | Item 6  Management of confounding factors | Item 7  Validity of measures | Item 8  Statistical analysis used |
| --- | --- | --- | --- | --- | --- | --- | --- | --- |
| Aoun, Cafarella, et al. (2021) | Yes | Yes | No | NA | No | NA | Yes | Yes |
| Aoun (2020) | Yes | Yes | Yes | NA | Yes | Yes | Yes | Yes |

*Note*. Item 1 = Were the criteria for inclusion in the sample clearly defined? Item 2 = Were the study subjects and the setting described in detail? Item 3 = Was the exposure measured in a valid and reliable way? Item 4 = Were objective, standard criteria used for measurement of the condition? Item 5 = Were confounding factors identified? Item 6 = Were strategies to deal with confounding factors stated? Item 7 = Were the outcomes measured in a valid and reliable way? Item 8 = Was appropriate statistical analysis used? NA = Not applicable.

**Supplementary Table 4**

*Quality rating of qualitative studies included in the review (Joanna Briggs Institute appraisal checklist for qualitative research)*

| Author(s)  (year) | Item 1  Philosophical premises | | Item 2  Objectives and study methodology | | Item 3  Data collection method | Item 4  Data analysis | Item 5  Interpretation of results | | Item 6  Researcher’ orientation | Item 7  Influence of the researcher | Item 8  Representation of participants | Item 9  Evidence of ethics | Item 10  Conclusions |
| --- | --- | --- | --- | --- | --- | --- | --- | --- | --- | --- | --- | --- | --- |
| Aoun et al. (2012) | | No | | Yes | Yes | Yes | | Yes | Yes | No | Yes | Yes | Yes |
| Aoun, Noonan, et al. (2021) | | No | | No | Yes | No | | No | No | No | Yes | Yes | Yes |
| Poppe et al. (2022) | | Unclear | | Yes | Yes | Yes | | Yes | Yes | No | Yes | Yes | Yes |
| Ray & Street (2007) | | No | | Yes | Unclear | Yes | | Yes | Yes | No | Yes | Yes | Yes |
| Ray et al. (2014) | | No | | Yes | Unclear | Unclear | | Yes | No | No | Yes | Yes | Yes |
| Solomon & Hansen (2015) | | Yes | | Yes | Yes | Yes | | Yes | Yes | No | Yes | Yes | Yes |
| Warrier et al. (2019) | | No | | Yes | Yes | Yes | | Yes | No | No | Yes | Yes | Yes |
| Whitehead et al. (2021) | | No | | Yes | Yes | Yes | | Yes | No | No | Yes | Yes | Yes |

*Note*. Item 1 = Is there congruity between the stated philosophical perspective and the research methodology? Item 2 = Is there congruity between the research methodology and the research question or objectives? Item 3= Is there congruity between the research methodology and the methods used to collect data? Item 4 = Is there congruity between the research methodology and the representation and analysis of data? Item 5 = Is there congruity between the research methodology and the interpretation on results? Item 6 = Is there a statement locating the researcher culturally or theoretically? Item 7 = Is the influence of the researcher on the research, and vice-versa, addressed? Item 8 = Are participants, and their voices, adequately represented? Item 9 = Is the research ethical according to current criteria or, for recent studies, and is there evidence of ethical approval by an appropriate body? Item 10 = Do the conclusions drawn in the research report flow from the analysis, or interpretation, of the data? Unclear = Not enough information provided to make the decision.
